# Exploring the Feasibility of a Self-Managed Lifestyle Intervention, Based on Exercise and Behaviour Support, as an Adjunct Therapy to Compression: A Sub-Study Focusing on People with Venous Leg Ulcers and Early Neuro-Degenerative Diseases (FISCU-NDD)

**DOI:** 10.3390/healthcare11202728

**Published:** 2023-10-13

**Authors:** Markos Klonizakis, Anil Gumber, Alexandra Morley, Michelle Horspool, Emma McIntosh, Maria Levesley, Jane McKeown, Pip Logan

**Affiliations:** 1 Lifestyle, Exercise and Nutrition Improvement (LENI) Research Group, Department of Nursing and Midwifery, Sheffield Hallam University, Sheffield S10 2BP, UK; a.gumber@shu.ac.uk (A.G.); e.mcintosh@shu.ac.uk (E.M.); 2 College of Health, Wellbeing and Life Sciences, Sheffield Hallam University, Sheffield S10 2BP, UK; a.morley@shu.ac.uk; 3 Sheffield Health & Social Care NHS Foundation Trust, Fulwood House, Old Fulwood Road, Sheffield S10 3TH, UK; michelle.horspool@shsc.nhs.uk (M.H.); jane.mckeown@shsc.nhs.uk (J.M.); 4 Integrated Care Team, Sheffield Teaching Hospitals NHS Foundation Trust, Lightwood House, Sheffield S10 2JF, UK; m.levesley@nhs.net; 5 Centre of Rehabilitation and Ageing Research, School of Medicine, Faculty of Medicine & Health Sciences, Nottingham NG7 2UH, UK; pip.logan@nottingham.ac.uk

**Keywords:** venous leg ulcers, dementia, Parkinson’s, lifestyle intervention

## Abstract

Background: The aim of this study was to adapt the “FISCU Home” intervention (a co-produced, self-managed and expert-supported lifestyle intervention comprising exercise and behaviour support aimed at people with Venous Leg Ulcers (VLUs), in a way that is suitable for the needs of people with combined VLUs and early-stage, Neuro-degenerative diseases (NDDs), and to explore its feasibility (e.g., estimate rates of recruitment and completion of sessions, calculate study adherence rates, assess participant satisfaction via participant interviews, and assess ease of data collection) within this clinical sub-group. Methods: We recruited seven people belonging to this VLUs sub-group (e.g., people with early-stage dementia or Parkinson’s), who were ≥18 years’ old, had VLU(s) of diameter ≥1 cm, ABPI ≥ 0.8, had the ability to tolerate lower-leg compression and were receiving VLU treatment at home. In Phase 1, participants helped us adapt the intervention. In Phase 2 we carried out a 4-week “training crash-course”. This consisted of three, 1 h, self-managed, exercise sessions per week (12 sessions in total), among the participants that completed the interviews. For Phase 3, we carried out post-interviews with all participants to investigate their study experiences, which were analysed using content analysis. Results: All assessments were completed successfully (100% retention and assessment completion), with no exercise-related adverse events. All participants completed the 4-week intervention (100%; all sessions completed by all participants). Conclusion: Our findings suggest that the adapted intervention is feasible, enjoyable and well-received, and has the potential to provide clinical benefits to the participants.

## 1. Introduction

Neuro-degenerative diseases (NDDs) is an umbrella term for conditions, which primarily affect the human brain neurons (i.e., dementias, Parkinson’s disease). Of these, dementias represent over 70% of cases, with 920,000 cases in the UK [1]. Thus, it is no surprise that almost 20% of those who receive home treatment for Venous Leg Ulcers (VLUs) (estimated at approximately 650,000 in the UK alone), have NDDs as well. The FISCU-II study [2] explored the feasibility of using “FISCU Home” (comprising a tailored exercise programme of aerobic, flexibility and resistance exercises with an embedded behaviour support programme delivered alongside compression), building on the experience of a successful group-based, lifestyle intervention [3]. Our FISCU-II study experience [4] suggests that people with early-stage NDDs were either reluctant to participate (i.e., afraid that they could not meet the study needs) or were not referred to the programme, despite the potential clinical benefits, because of their condition. Consequently, in order to overcome the participation challenges faced by people with NDDs, we decided to carry out a small-scale study (FISCU-NDD), engaging with this VLUs sub-group (e.g., people with early-stage dementia or Parkinson’s).

FISCU-NDD aimed at: (a) adapting the “FISCU Home” lifestyle intervention [4] in a way that is suitable for the needs of people with VLUs and NDDs, and (b) exploring its feasibility (e.g., estimating rates of recruitment and completion of sessions, calculating study adherence rates, exploring participant experiences via personal interviews, and assessing ease of data collection) within this sub-group. Collecting this information would allow us to explore its efficacy, in a larger study.

## 2. Methods

We recruited people belonging to this VLUs sub-group (e.g., people with early-stage dementia or Parkinson’s), who also complied with the original FISCU-II study criteria (e.g., ≥18 years’ old, VLU(s) of diameter ≥1 cm, ABPI ≥ 0.8, ability to tolerate lower-leg compression and receiving treatment at home), and their carers, in a three-phase study in order to further adapt our “FISCU Home” intervention. This study was ethically approved and adhered to the guidelines set out in the Declaration of Helsinki. Potential participants were identified by the Integrated Care Team of Sheffield Teaching Hospitals NHS FT. Nineteen people were approached; ten participants agreed to participate (53%), while seven were invited to be recruited, and consequently consented and were interviewed (Table 1).

### 2.1. Phase 1: Adaptation

In Phase 1, participants helped us adapt the “FISCU Home” intervention. Adaptations were made to the wording and design of the support documentation (i.e., participant information sheet, exercise manual, resources use diary), the support sessions’ structure (i.e., a special “session objectives reminder” element was introduced at each session’s start), the inclusion of an invitation to carers to participate at introductory sessions and the choice of exercises used (with the original extended range being reduced to avoid confusion and more complex exercises being removed as well).

### 2.2. Phase 2: Feasibility/Pilot

In Phase 2, we carried out a 4-week “training crash-course”. This consisted of three 1 h, self-managed, exercise sessions per week (12 sessions in total; 1), among the participants that completed the Phase 1 interviews. Training was based around a series of aerobic exercises (e.g., step-ups, walking a figure of eight, etc.). Flexibility (e.g., hamstring stretches, ankle stretches) and resistance exercises (e.g., knee extensions, standing calf raise) were also included. All exercises were selected in Phase 1 by study participants, as appropriate for this clinical group. An intervention facilitator supported the delivery of the intervention via face-to-face visits at the participants’ residences and phone calls (e.g., by setting up and adapting the exercise programme when necessary, monitoring their progress and fidelity and delivering the behaviour support element of “FISCU Home”). The latter included motivating the participants (and their carers) to continue being engaged with the intervention, ways to adapt their lifestyle and house environment to achieve this and general healthy lifestyle advice, among others.

All participants received standard compression therapy via regular (e.g., weekly) visits carried out by experienced tissue viability-trained nurses, aiming for 40 mmHg of pressure at the ankle, which graduated to 17–20 mmHg at the upper calf.

We carried out two assessment days (pre- and post-intervention), collecting ulcer-related data, anthropometrics, baseline exercise and medical history, quality of life and physical fitness (via the Senior Fitness Test (2 min walking test, chair sit and stand) indices and assessing ankle range of motion.

Feasibility data were also collected. Our progression criteria included: (i) At least 35% of eligible, approached patients being willing to be randomised, (ii) at least 67% of randomised patients in the exercise groups being compliant (e.g., ≥75% of scheduled sessions are completed), (iii) loss to follow-up at 4 weeks being less than 20% and (iv) the absence of exercise-related adverse events during the intervention sessions.

### 2.3. Phase 3: Qualitative Process Evaluation

For Phase 3, we carried out post-intervention interviews with all study participants in the presence of their carers, where applicable, to explore their intervention experiences, as well as their views on their treatment. These were analysed using content analysis, following standard practice [5].

## 3. Results

### 3.1. Feasibility Outcomes

All assessments were completed successfully (100% retention and assessment completion). There were no exercise-related adverse events, and all participants completed their treatment course as prescribed and instructed. All participants completed the 4-week intervention (100%; all sessions completed by all participants); however, only 20% of the sessions were completed independently (e.g., without support), with a median of six weeks needed due to illnesses/personal issues: the rest required the support of carers or the intervention facilitator.

### 3.2. Physical Fitness, Ulcer-Related and Quality of Life Outcomes

There were no changes in calf and arm circumferences. In contrast, physical fitness indices were improved at the end of the intervention (i.e., 17 (0–40) vs. 13 (0–30) for the two-minute steps). All but one participant had their ulcer healed, with the mean ulcer size for the group being reduced. No recurrent ulcers were reported at the end of the intervention. In a similarly positive outcome, study participants experienced an increase in their overall quality of life at the end of the intervention (e.g., 63 (40–80) vs. 55 (30–75). All outcomes are presented in Table 2.

### 3.3. Qualitative Outcomes

Similar to the general VLUs population [6], participants enjoyed the interaction and found the study useful. The exercises were perceived to be easy, while some participants felt the hardest aspect was remembering to do the exercises. Participants felt that benefits were not just focused on ulcer healing, but included improved mobility, a sense of achievement, increased confidence and pain reduction.

## 4. Discussion

Worldwide, it is estimated that at least one-third of NDD cases are related to lifestyle factors, one of which is limited physical activity [7]. Therefore, it is possible that an increase in physical activity levels will result in improvements in both general health and cognitive functioning in people with NDDs. Further potential benefits may be prominent for people with VLUs with NDDs, including an improved VLUs healing perspective as well [8]. Despite the great potential, certain barriers exist that prevent people with NDDs from participating in exercise-based, lifestyle interventions, including behavioural and psychological symptoms of dementia (e.g., apathy), low motivation (e.g., the belief that exercise will not help either condition) and/or fear of exercise. Our study is the first to co-produce, with people with combined VLUs and NDDs, a tailored, lifestyle intervention intended to be used alongside compression to treat VLUs. Our findings suggest that the adapted intervention is feasible, enjoyable and well-received, having the potential to bring clinical benefits to the participants (Table 2).

However, it becomes clear that due to the nature of the co-morbidity, completing the sessions independently, can be challenging for people with NDDs. The role of carers has previously been recognized in the success of any NDD-targeting intervention [9,10]; our study has recognized this need and thus, carers have provided support in the co-production stage of our sub-study. Nevertheless, it becomes clear that carers should be actively involved at the delivery stage as well, an approach that was proven to be successful both in the present study, as well as in previous ones i.e., [9]. Further initiatives (such as encouraging carers to participate in the intervention as well, reminding them about the potential benefits of exercise, etc.) could potentially increase motivation and should be implemented. Equally, an increase in training sessions supported by an intervention facilitator should also be considered.

### Limitations

This study aimed to direct future research and explored the potential for adapting and (consequently) implementing a home-based lifestyle intervention (e.g., “FISCU Home”) as an adjunct therapy, alongside compression among people with VLUs and NDDs. Therefore, as we recruited only a limited number of participants, our clinical findings should be treated with caution. Moreover, our aim was to carry out a short-term intervention, which will act as a prelude to long-term ones; this means that clinical findings may differ if “FISCU Home” is followed for a longer period. Nevertheless, the great potential of “FISCU Home” is evident.

## 5. Conclusions

Our study supports the notion that “FISCU Home”, a lifestyle intervention based on aerobic exercise and behaviour support that aims to assist participants in adapting to a healthier lifestyle, has the potential to assist people with VLUs and NNDs in their treatment journey. Nevertheless, further adaptations are needed, including potentially an increased number of supported sessions and greater involvement of their carers (where applicable). These should be considered in any future interventions targeting this sub-group of the VLUs clinical population, before any wider implementation.

## Figures and Tables

**Table 1:** Sub-study participants’ baseline profile.

Baseline Characteristics	Mean (Range)
1. Male gender, *n*	1/7 (14%)
2. Age in years, mean (Range)	84 (67–92)
3. Working, *n* (%)	0/7 (0%)
4. White ethnicity, *n* (%)	7/7 (100%)
5. Stature (cm), mean (Range)	150 (145–157)
6. Body mass, mean (Range)	76 (53–129)
7. Smoking status—Previous	1/7 (14%)
8. Alcohol consumption	0/7 (0%)
9. Diagnosis	Parkinson’s = 3
	Dementia = 4
10. Number of co-morbidities reported, mean (SD)	4 (3–6)
11. Number of prescribed medications, mean (SD)	8 (3–15)
**Ulcer Related**	
1. Duration of reference ulcer, mean months (SD)	7 (0–16)
2. Time since diagnosis of reference ulcer, mean months (SD)	5 (2–9)
3. ABPI, mean (SD)	1.1 (0.9–1.2)
**Physical Activity and Fitness**	
1. Walking Pace—slow pace, *n* (%)	7/7 (100%)
3. Unable to do housework/childcare, *n* (%)	6/7 (86%)
4. Driving Before—Don’t Drive	6/7 (86%)
5. Driving After Ulcer—Don’t Drive	7/7 (100%)

**Table 2:** Fitness-, ulcer- and quality-of-Life-related data. Mean (range) data are shown.

Physical Fitness Assessment	Baseline	1 Month
1. Flexibility, mean (°)	−21 (−39 to 0)	−18 (−10 to −28)
2. Angular Plantar, mean (°)	4 (3–8)	7 (5–11)
3. Angular Dorsi, mean (°)	12 (5–20)	14 (2–22)
4. Ankle Range, mean (°)	17 (13–25)	20 (7–28)
5. Ankle circumference (cm)	22 (20–27)	21 (19–26)
6. Calf circumference (cm)	36 (26–53)	36 (27–56)
7. Two-minutes steps (number)	13 (0–30)	17 (0–40)
8. Chair sit-to-stand (repetitions)	1 (0–3)	2 (0–3)
**Ulcer Related Assessments**	**Baseline**	**1 Month**
Participants with active ulcers (*n*)	*n* = 7	*n* = 1
Length (cm)	2.5 (1–7)	1
Width (cm)	1.6 (0.8–3.5)	1.5
Area (sq. cm)	6.1 (1–24.5)	1.3
Recurrence	N/A	*n* = 0
**Quality of Life Assessments**	**Baseline**	**1 Month**
Mobility	4 (4–4)	4 (4–4)
Self-care	4 (3–5)	4 (4–4)
Usual activity	4 (3–5)	4 (3–5)
Pain	3 (2–4)	3 (2–5)
Anxiety/Depression	2 (2–3)	3 (2–3)
Quality-Adjusted Life Years (QALY)	0.27 (0.09–0.38)	0.26 (0.02–0.40)
Quality of Life Visual Analogue Scale (0–100)	55 (30–75)	63 (40–80)

QoL Levels: 1—No problem, 2—Mild, 3—Moderate, 4—Severe, 5—Extreme/Unable to do.

## Data Availability

Data are unavailable due to privacy and ethical constraints.
